# Adaptive *dif* Modules in Permafrost Strains of *Acinetobacter lwoffii* and Their Distribution and Abundance Among Present Day *Acinetobacter* Strains

**DOI:** 10.3389/fmicb.2019.00632

**Published:** 2019-03-29

**Authors:** Sofia Mindlin, Alexey Beletsky, Andrey Mardanov, Mayya Petrova

**Affiliations:** ^1^Laboratory of Molecular Genetics of Microorganisms, Institute of Molecular Genetics, Russian Academy of Sciences, Moscow, Russia; ^2^Laboratory of Microorganism Genomics and Metagenomics, Institute of Bioengineering, Research Center of Biotechnology of the Russian Academy of Sciences, Moscow, Russia

**Keywords:** plasmid p*dif* sites, additional chromosomal *dif* sites, *terC dif*, *ohr1 dif*, *sulP dif*, *kup dif*, *add dif*

## Abstract

The *dif*/Xer system of site-specific recombination allows resolution of chromosomal dimers during bacterial DNA replication. Recently, it was also shown to be involved in horizontal transfer of a few known Xer-dependent mobile elements. Here, we show that plasmids of various *Acinetobacter* species, including clinically important strains, often contain multiple p*dif* sites that are mainly located within their accessory regions. Chromosomes of *Acinetobacter* strains may also contain additional *dif* sites, and their similarity with plasmid p*dif* sites is higher than with the main chromosomal site *dif1*. We further identify putative mobile genetic elements containing p*dif* sites on both flanks of adaptive genes and analyze their distribution in *Acinetobacter* species. In total, we describe seven mobile elements containing genes with various adaptive functions from permafrost strains of *A. lwoffii* group. All of them are also spread in modern plasmids of different *Acinetobacter* species including *A. baumannii*. We could not detect p*dif* sites and corresponding mobile elements in closely related bacterial genera, including *Psychrobacter* and *Moraxella*. Thus, the widespread distribution of *dif* modules is a characteristic feature of *Acinetobacter* species and may contribute to their high adaptability both in the environment and in the clinic.

## Introduction

*Acinetobacter* strains are common everywhere due to their ability to adapt to various environments (Touchon et al., 2014). Many of them – first of all, *Acinetobacter baumannii*, but also *Acinetobacter nosocomialis*, *Acinetobacter pittii*, *Acinetobacter lwoffii*, *Acinetobacter ursingii*, *Acinetobacter haemolyticus*, *Acinetobacter parvus*, *Acinetobacter junii* – are among the most important pathogens in the clinic (Turton et al., 2010; Peleg et al., 2012; Evans and Amyes, 2014; Touchon et al., 2014). The rise of antimicrobial resistance in these strains is a burning medical problem, with a large number of mobile genetic elements involved in the spread of the resistance determinants (Martins et al., 2015; Da Silva and Domingues, 2016; Pagano et al., 2016).

Recently a novel class of mobile genetic elements whose transposition likely depends on the action of the *dif/*Xer recombination system was discovered in *Acinetobacter* species. Initially, a discrete DNA module that contained the gene encoding OXA-24 carbapenemase, flanked by conserved inverted repeats homologous to the XerC/XerD binding sites (*dif* sites), was revealed in an *A. baumannii* plasmid pABVA01a. It was shown that this element (later designated *dif* module) is also present in a different context in another *Acinetobacter* plasmid, suggesting its horizontal transfer (D’Andrea et al., 2009). Similar observations were soon made by other laboratories when studying plasmids containing the same gene OXA-24 (Merino et al., 2010; Grosso et al., 2012) and related carbapenemase genes OXA-72 (a variant of OXA-24) or OXA-58 (Povilonis et al., 2013; Blackwell and Hall, 2017). Subsequent studies demonstrated that other antibiotic resistance genes can also be included in *dif* modules. In particular, Blackwell and Hall (2017) described two novel *dif* modules containing genes encoding resistance to tetracycline and erythromycin.

The *dif* sites found in *Acinetobacter* plasmids (p*dif* sites) are similar to the chromosomal *dif1* site recognized by the dif/Xer system of site-specific recombination, which plays an essential role in the resolution of chromosomal and plasmid dimers (Carnoy and Roten, 2009; Castillo et al., 2017). Orphan p*dif* sites could also be revealed in a number of plasmids (Carnoy and Roten, 2009; Blackwell and Hall, 2017; Cameranesi et al., 2018), and the reaction of site-specific recombination could be observed between the two sites present in different plasmids, suggesting that this system is involved in the horizontal transfer of *dif* modules (Cameranesi et al., 2018).

Further studies have shown that the spread of *dif* sites and *dif* modules is not limited to plasmids of clinical *Acinetobacter* strains and that they can include a variety of genes, not only determinants of antibiotic resistance. In particular, we recently described a novel *dif* module containing chromium resistance genes (*chrAB dif*). Initially discovered in plasmids from ancient (permafrost) strains of *A. lwoffii* it was also found in plasmids of modern *Acinetobacter* strains (Mindlin et al., 2018). We also identified two other potential *dif* modules, *kup dif* and *sulP dif*, but did not characterize them in detail (Mindlin et al., 2018). Recently, putative new *dif* modules containing genes with potential adaptive functions were found in plasmids of the clinical isolate of *A. baumannii* D36 (Hamidian and Hall, 2018). This suggested that formation of mobile elements flanked by p*dif s*ites and carrying various adaptive genes may be a characteristic feature of plasmids of the whole *Acinetobacter* genus.

In the present work, we investigated the distribution of *dif* sites in more than 180 *Acinetobacter* plasmids belonging to different species. The main emphasis was made on the identification and analysis of *dif* modules from ancient *A. lwoffii* plasmids and studies of their distribution in modern strains. As a result, we identified four novel adaptive *dif* modules, in addition to *chrAB dif*, *kup dif*, and *sulP dif*, and revealed their broad distribution in plasmids in environmental and clinical strains of the *Acinetobacter* genus. We also showed that most *Acinetobacter* chromosomes contain additional *dif* sites besides the main site *dif1*, which may contribute to the distribution of *dif* modules in this genus.

## Materials and Methods

### Bacterial Strains and Growth Conditions

*Acinetobacter* strains ED23-35, ED45-23, ED9-5a, EK30A, and VS15 used in this study were previously isolated from 15 thousand to 3 million years old permafrost sediments collected from different regions of Kolyma Lowland. We here in referred to these strains as “ancient” strains, which never come into contact with clinical isolates. Initially they were identified as *A. lwoffii* based on metabolic testing and from 16S rRNA gene sequence analysis (Petrova et al., 2002; Kurakov et al., 2016; Mindlin et al., 2016). In this work, based on data from a recent article by Nemec et al. (2018), revising the taxonomy of the *Acinetobacter lwoffii* group we showed that strain ED9-5a actually belongs to the *Acinetobacter pseudolwoffii* species. The remaining permafrost strains do belong to *A. lwoffii*. In addition, we analyzed two sets of *Acinetobacter* strains obtained from publicly available databases (“modern” strains). These were (i) 38 strains of *A. baumannii* described by Shintani et al. (2015), and (ii) 30 strains belonging to other *Acinetobacter* species obtained from public databases before January 2018. Bacteria were grown in lysogeny broth (LB) medium or solidified LB medium (LA) at 30°C.

### Confirmation of the Species Identification and Identification of Misidentified Isolates

Species identification was confirmed by comparison of partial *rpoB* gene sequences with the reference *Acinetobacter* genomes^1^. The *rpoB* gene nucleotide identity level of 98–100% was regarded as evidence that the compared genomes belong to the same species.

### Whole-Genome Sequencing and Assembly of Plasmids

The genomes were sequenced with a Roche GS FLX genome sequencer (Roche, Switzerland) using the Titanium protocol to prepare shotgun genomic libraries. The GS FLX reads were assembled into contigs using the GS De Novo Assembler (Roche); protein coding genes were identified and annotated using the RAST web server. To identify potential plasmid contigs we used read coverage depth and annotation information, such as existence of genes for mobilization and/or replication of plasmids. Using 454 assembly graph file (output of the GS De Novo Assembler) we extended and merge our potential plasmid contigs with each other. Some additional merging of contigs was done using PCR. All manually assembled regions were checked using PCR. A plasmid was considered as finished if it was circular according to the 454 assembly graph and PCR check. The 454 reads were mapped back to the finished sequences using 454 GS Reference Mapper, and read mapping was visualized in IGV browser and manually inspected for potential missassemblies.

### Screening of *Acinetobacter* Plasmids and Chromosomes for the Presence of *dif* Sites

A collection of plasmids isolated from different *Acinetobacter* species (Supplementary Table S1) was screened for the presence of p*dif* sites by BLAST software. The XerC/XerD (3916–3943 bp) and XerD/XerC (6899–6926) p*dif* sites of plasmid pM131-6 [NC_025120] were used as a reference. Only sites that were at least 75–80% identical to the reference were taken into account. The same algorithm was used in the search for the main and additional *dif* sites in *Acinetobacter* chromosomes.

### Bioinformatics Analysis

For the assembly and analysis of plasmid genomes from ancient strains, the program UGENE^2^ was used. Similarity searches were performed using BLAST (Altschul et al., 1997) and REBASE (Roberts et al., 2009). Conserved domains and motifs were identified using the NCBI Conserved Domain Database (CDD) (Marchler-Bauer et al., 2011) and the Pfam database (Finn et al., 2009). Dif sites were clustered using blastclust from blast package with 95% identity threshold. Fasta files with dif sites were sorted according to blastclust results using custom perl script (dif sites from the same cluster follow each other, and clusters are sorted by size). The sorted fasta files were visualized in AliView as color blocks. The fasta files with dif sites were used to create sequence logos using WebLogo^3^ (Crooks et al., 2004).

Identification of *dif* modules in permafrost plasmids and determination of their mobility was carried out manually in several stages. Initially, plasmids containing at least two different p*dif*-sites were selected. Then, the regions between the pairs of p*dif*-sites were analyzed, and, based on the presence of a potentially adaptive gene (s) in this region candidates for mobile modules were revealed. At the final stage, using the Blast software, the distribution of the identified modules among the ancient (isolated from permafrost) and modern strains of *Acinetobacter*’species was investigated. The presence of the same module with an identity level of 98–99% in different regions of different plasmids (contigs) isolated from *Acinetobacter* strains belonging to different species was regarded as evidence of its mobility.

### Determination of the MIC for Tellurium and Organic Peroxide Resistance

The level of resistance was determined by the agar dilution method (Mindlin et al., 2016). Overnight cultures of bacterial strains were diluted 10-fold with fresh LB and grown at 30°C with shaking for 3–4 h; the cultures were then induced for 1 h by the addition of tert-butyl hydroperoxide (tBHP) at 0.1 mM. Five μl of the bacterial suspension (about 5 × 10^6^ cells per ml) were plated onto LA supplemented with Na_2_TeO_3_ (Tel) or tBHP. The concentrations tested were as follows: Tel – 0.01; 0.02; 0.04 and 0.1 mg/ml and tBHP – 0.05; 0.1; 0.2; 0.3 and 0.4 mM. The plates were incubated at 30°C for 24 h and visually inspected. In each case, three independent experiments were performed.

### Nucleotide Sequence Accession Numbers

The accession numbers for nucleotide sequences of the plasmids deposited in this work in the GenBank database are as follows: CP032112.1 (pALWED1.2); CP032113-CP032116.1 (pALWED1.4-1.7); CP032117.1-CP032124.1 (pALWED2.2-2.9); CP032287.1-CP032289.1 (pALWED3.2-3.4); CP032290.1 (pALWED3.6); CP032102.1 (pALWEK1.1); CP032105.1-CP032107.1 (pALWEK1.2-1.4); CP032108.1- CP032111.1 (pALWEK1.6-1.9); CP032103.1 (pALWEK1.10); CP032104.1 (pALWEK1.11).

## Results

### Distribution of p*dif* Sites in Plasmids From Strains of Different *Acinetobacter* Species

For analysis of the occurrence of *pdif* sites in *Acinetobacter* plasmids, we studied plasmids found in strains of various *Acinetobacter* species, divided into three groups (see Materials and Methods): (1) plasmids of permafrost strains of *A. lwoffii* and *A. pseudolwoffii* isolated and studied in our group (35 plasmids, see below) together with plasmids from *A. lwoffii* ZS207 (10 plasmids, see Supplementary Table S1); (2) plasmids from *A. baumannii* strains, described in Shintani et al. (2015) (65 plasmids, Supplementary Table S1); (3) plasmids isolated from strains belonging to others *Acinetobacter* species available from public databases before January 2018 (73 plasmids, Supplementary Table S1).

**FIGURE 1:**
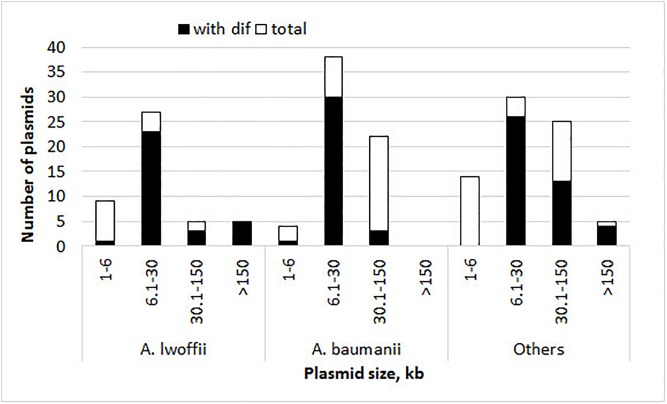
Histograms of plasmid size and their numbers with and without p*dif* sites. The height of the column indicates the total number of plasmids, and its black part shows the number of plasmids containing p*dif* sites.

The presence of p*dif s*ites and the number of their copies in each plasmid were determined by the BLAST tool. The results obtained for each set of plasmids are presented in Figure 1, Table 1, and Supplementary Table S1. Plasmid p*dif* sites were found in more than 50% of the *Acinetobacter* plasmids in all three groups (Table 1). In most cases, the number of sites was 1–4, while some plasmids contained 5–8 copies of p*dif* or more (Table 1). Most p*dif* sites were found in small to middle size plasmids (6–30 kb), while very small plasmids (<6 kb) as a rule did not contain them.

**Table 1:** Distribution and abundance of p*dif* sites in *Acinetobacter* plasmids.

Species	Number (%) of plasmids with a given number of p*dif* sites in one plasmid				Total number of plasmids
	0 p*dif*	1–4 p*dif*	5–8 p*dif*	>8 p*dif*	
*A. baumannii*	31 (47,7)	28 (43,1)	4 (6,1)	2 (3,1)	65
*A. lwoffii* and	13 (28,9)	23 (51,1)	3 (6,7)	6 (13,3)	45
*A. pseudolwoffii*					
other species	30 (41,1)	28 (38,3)	12 (16,4)	3 (4,1)	73
Total	74 (40,4)	79 (43,2)	19 (10,4)	11 (6,0)	183

Interestingly, multiple copies of *pdif* sites were found both in very large and in middle-size plasmids (22–400 kbp) (Figure 1 and Table 2).

**Table 2:** List of plasmids containing multiple p*dif* sites (>10).

Strain	Plasmid	Length, bp	Number of p*dif* sites		Accession No.
			C/D	D/C	
*A. lwoffii* ED45-23	pALWED2.1	191,611	8	8	KX426229
*A. lwoffii* ED45-23	pALWED2.3	22,771	6	5	CP032118.1
*A. lwoffii* EK30A	pALWEK1.1	209,982	7	5	KX528688
*A. lwoffii* ZS207	pmZS	198,391	9	14	CP019144.1
*A. baumannii*	pABIR	29,823	6	5	NC_010481
*A. johnsonii* XBB1	pXXB1-9	398,857	7	7	CP010351.1
*A. pittii* AP_882	pOXA58-AP_882	36,862	6	7	CP014479.1
*A*. sp NCu2D-2	Unnamed	309,964	6	7	CP015595.1

**FIGURE 2:**
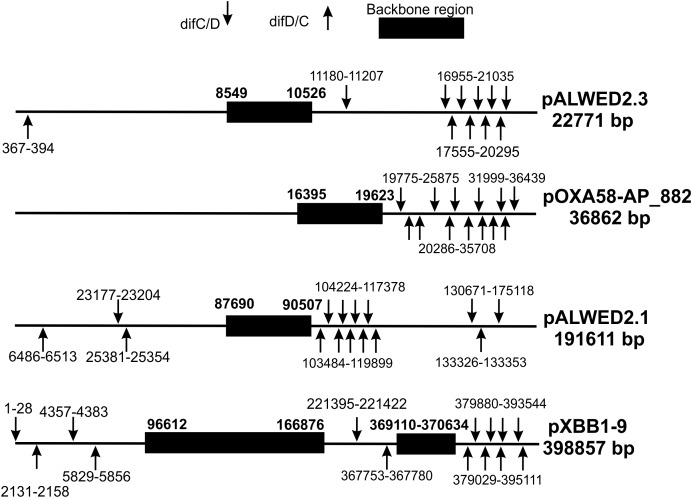
Distribution of p*dif* sites over the plasmid genomes (not to scale).

To determine the localization of p*dif* sites relative to the backbone and accessory plasmid regions, we analyzed their distribution in four plasmids, two middle size (pALWED2.3 and pOXA58-AP_882) and two large (pALWED2.1 and pXBB1-9), each containing more than 10 sites (Table 2). For all four plasmids, *pdif* sites were distributed unevenly across the plasmid sequence; notably, they were absent from the backbone regions and formed clusters in certain plasmid regions (Figure 2).

To our knowledge, there is currently no data on the distribution of typical p*dif* sites in the plasmids of bacteria from other systematic groups. To get insight into their abundance in closely related genera to *Acinetobacter*, we analyzed a total of 31 plasmids from 16 strains of *Psychrobacter* (2117–44793 bp) and 8 plasmids from 6 strains of *Moraxella* (1313–44215 bp) described by Shintani et al. (2015) and Moghadam et al. (2016). No p*dif* sites were detected in any of these plasmids. Therefore, the presence of a large number of p*dif* sites may be a unique feature of *Acinetobacter* plasmids.

### Identification of *dif* Modules in Plasmids From Permafrost *A. lwoffii and A. pseudolwoffii* Strains

The primary search for *dif* modules was performed on the set of plasmids from five permafrost strains from our collection (ED23-35, ED45-23, ED9-5a, EK30A and VS15; Supplementary Table S1) (Petrova et al., 2002; Mindlin et al., 2016). In five fully sequenced genomes, a total of 35 plasmids of various sizes were found. In addition to previously described 8 plasmids (Mindlin et al., 2016, 2018), we have now assembled 27 new plasmids from the same strains (see details in Materials and Methods).

In 10 of the plasmids, sequences homologous to p*dif* sites were absent or only a single p*dif* site was detected (Supplementary Table S1). The remaining 25 plasmids with two or more p*dif* sites were analyzed for the presence of *dif* modules containing adaptive genes surrounded by two *pdif* sites (see Materials and Methods). In total, putative *dif* modules were detected in 12 plasmids; 6 of them contained one module; 4 contained two modules, and 2 contained three different modules (Supplementary Table S2).

**FIGURE 3:**
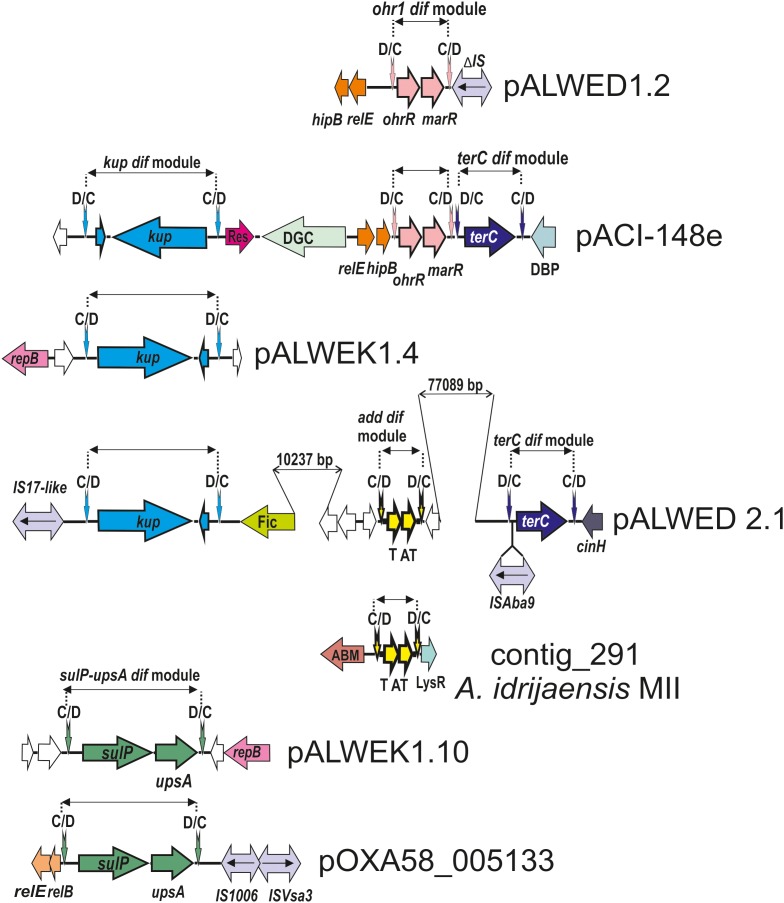
Comparative genetic structures of plasmid regions containing *dif* modules. The locations and directions of genes and ORFs are shown by arrows. The components of the *dif* modules are represented by arrows with different colors. Vertical arrows indicate p*dif* sites with the orientation of the subsites shown above. Numbers (bp) show the size of the sequences located between different genes. Designations of gene products are as follows: DBP, DNA-binding protein; DGC, diguanylate cyclase; Res, resolvase; T, toxin; AT, antitoxin; Fic, Fic/DOC family protein; ABM, antibiotic biosynthesis monooxigenase; LysR, LysR family transcriptional regulator; unnamed ORFs, hypothetical proteins. Designations of IS-s: ISVsa3 -100% with ISVsa3; IS1006 – 1 mismatch with IS1006; ISAba9 – 2 mismatches with ISAba9; IS17-like -96% identity with IS17; ΔIS – truncated copy of an IS5 family element (IS427 group). The picture is drawn to scale.

In addition to the three modules identified previously (*chrAB dif*, *kup dif*, and *sulP dif*) (Mindlin et al., 2018), we revealed four new putative modules with adaptive genes: *terC dif*, *add dif*, *ohr dif*, and *sulP-uspA dif* (Figure 3).

In most cases, plasmids containing the same modules originated from different permafrost strains (Supplementary Table S2) suggesting their horizontal transfer. To test this hypothesis, we analyzed the distribution of all modules among plasmids (contigs) of modern *Acinetobacter* strains belonging to different *Acinetobacter* species (Supplementary Table S2). Below, we briefly describe characteristics and distribution of each of these modules (Table 3).

**Table 3:** The *dif* modules found in permafrost *A. lwoffii* strains and their distribution in modern strains.

Module	Length (b.p.)	Proposed function	Number of copies in plasmids/contigs	
			Environmental strains	Clinical strains
*chrAB dif*	3011	Resistance to chromium	9	2
*terC dif*	1221	Resistance to tellurium	8	1
*kup dif*	2549	Potassium uptake	7	0
*ohr1 dif*	1063	Resistance to organic peroxides	2	2
*sulP dif*	2159	Sulfate transporter	3	3
*sulP-uspA dif*	2739	Sulfate transporter	2	4
*add dif*	768	Toxin-antitoxin protein pair	5	3

### *terC dif* Module

We revealed the *terC dif* module (1221 bp) in four permafrost plasmids, pALWED1.4 from *A. lwoffii* ED23-35, pALWED2.1 from *A. lwoffii* ED45-23, pALWED3.6 from *A. pseudolwoffii* ED9-5a and pALWEK1.1 from *A. lwoffii* EK30A (Supplementary Table S2). It contained a single orf, identified as *terC* (978 bp), encoding resistance to tellurium. We also found the *terC dif* module in two modern plasmids and several contigs from modern strains belonging to different species of *Acinetobacter* (Supplementary Table S2 and Figure 3).

Up to date, genetic determinants of resistance to tellurium were found in the genomes of different bacteria both in chromosomes and plasmids (Chasteen et al., 2009). We also detected the *terC* gene in the sequenced chromosomes of *Acinetobacte*r strains, belonging to different species including *Acinetobacter baylyi* ADP1 [NC_005966.1] widely used in genetic studies.

However, the plasmidic and chromosomal *terC* genes were related only distantly (42–60% identity at the protein sequence level), and the chromosomal *terC* gene was not flanked by *dif* sites.

To determine the functional activity of the *terC di*f module, we compared the level of tellurium resistance of the permafrost strains of *A. lwoffii* and *A. pseudolwoffii* containing the *terC dif* module with the strain *A. lwoffii* VS15 that lacked this module in its plasmids. The results of three independent experiments showed that strains ED23-35, ED9-5a and EK30A have a significantly higher resistance level (MIC from 0.04 to 0.1 mg/ml) compared to strain VS15 (MIC < 0.01 mg/ml); whereas strain ED45-23, characterized by very slow growth, does not differ from VS15. Thus, additional studies are needed to confirm the contribution of the *terC dif* module into the tellurium resistance of corresponding host strains.

### *add dif* Module

Most prokaryotic chromosomes as well as many plasmids contain toxin-antitoxin (TA) systems consisting of a pair of genes that encode 2 components, a stable toxin and its cognate unstable antitoxin. TA systems are also known as addiction modules (Yamaguchi et al., 2011; Schuster and Bertram, 2013). In particular, many type II toxins are mRNA-specific endonucleases that arrest cell growth through RNA cleavage, thus preventing the process of translation (Bertram and Schuster, 2014). We revealed a number of type II TA modules in permafrost plasmids. Amongst them we found two related modules surrounded by p*dif* sites (both 768 bp long). Both modules contained two genes, one (297 bp) encoding a killer protein and another (291 bp) – an antidote protein. The first module was located in large plasmid pALWED2.1; the second was detected in medium-sized (22771 bp) plasmid pALWED2.3 and large plasmid pALWEK1.1. The difference in nucleotide sequences of the modules was 4%.

We also found variants of both *add dif* modules in plasmids and contigs of modern strains belonging to different *Acinetobacter* species (*A. lwoffii, A. baumannii, Acinetobacter towneri, Acinetobacter johnsonii)* (Supplementary Table S2). In most cases they were almost identical to the basic prototype modules (96–99% nucleotide identity level).

### *ohr dif* Module

This genetic element contains two genes: a gene encoding organic hydroperoxide resistance, *ohr* (429 bp) and a second gene *ohrR* (432 bp) encoding transcription regulator from the MarR family of transcription factors (Sukchawalit et al., 2001; Atichartpongkul et al., 2010).

The *ohr dif* module was detected in plasmids from three permafrost strains *A. lwoffii*: pALWED1.2 from strain ED23-35; pALWVS1.1 from strain VS15 and pALWEK1.1 from strain EK30A (identity level 99%). However, the size and the boundaries of this module differed between the plasmids. We therefore analyzed its structure and distribution in more detail.

The *ohr/ohrR* genes with plasmid localization were first found by Dorsey et al. (2006) in the plasmid pMAC [AY541809.1] from a reference clinical strain *A. baumannii* ATCC 19606. The authors showed that *A. baumannii* ATCC 19606 is resistant to the organic peroxide-generating compounds cumene hydroperoxide (CHP) and tert-butyl hydroperoxide (t-BHP). We analyzed the genome of pMAC and detected typical p*dif* sites on both flanks of the *ohr* genes suggesting that pMAC contains an *ohr dif* module. The permafrost and clinical versions of the *ohr dif* module are overall closely related (96% nucleotide identity) but reveal some specific differences: (*i*) the *ohr* module from the modern strain is 5 bp longer than the ancient one (1068 bp vs.1063 bp); (*ii*) both modules contain identical *ohrR* genes but their *ohrA* genes differ by 3%; (*iii*) region adjacent to the XerD/XerC site contains multiple substitutions. We therefore designated the ancient variant *ohr1 dif* and the modern variant *ohr2 dif* and studied the distribution of each of them separately.

Although we did not find the *ohr1 dif* module in the plasmids of modern strains, it was revealed among unassembled genomic sequences of various *Acinetobacter* species, in most cases in small-size contigs (Supplementary Table S2). The *ohr2 dif* module was detected both in plasmids and in contigs from various *Acinetobacter* species (Supplementary Table S2).

Interestingly, two plasmids (plasmid unnamed 2 [CP027180] and plasmid unnamed 4 [CP027186] from the modern strains of *A. baumannii* AR_0070 and *A. baumannii* AR_0052, respectively) contained numerous copies of the *ohr* 2 module, 14 in the first plasmid and 8 in the second.

The location of both *ohr dif* modules in various sequence contexts in different plasmids and contigs (Figure 3) strongly suggests their ability to move. Both modules likely originated from a common ancestor and then distributed independently among *Acinetobacter* species.

To test the functional activity of the *ohr1 dif* module, we compared resistance of permafrost strains, containing (group 1: ED23-35, VS15 and EK30A) or lacking (group 2: ED45-23 and ED9-5a) this module, to tert-butyl hydroperoxide (t-BHP) (see Materials and Methods). The results of three independent experiments demonstrated a higher level of resistance of group 1 strains (MIC = 0.2–0.4 mM) in comparison with group 2 strains (MIC = 0.1 mM).

### *dif* Modules Containing Gene *sulP*

The gene *sulP* encodes sulfate permease (484 aa) that perform transport of inorganic anions into cells (Price et al., 2004). It is a member of the large and ubiquitous family of SulP proteins present in archaea, bacteria, fungi, plants and animals (Alper and Sharma, 2013).

We revealed two different mobile elements containing the *sulP* gene. The first module *sulP dif* (2159 bp), first found in our previous work (Mindlin et al., 2018), contained a small orf (198 bp) encoding hypothetical protein in addition to *sulP*; the second module *sulP-uspA dif* (2739 bp), detected in this work in the plasmid pALWEK1.10 from *A. lwoffii* EK30A, contained the *uspA* gene encoding a universal stress protein in addition to *sulP*. The *sulP* genes from the two modules are related (the identity level of 75%). The distribution of both *dif* modules in modern plasmids (contigs) is presented in Supplementary Table S2 and Figure 3 illustrates the location of the *sulP-uspA dif* module in different plasmids.

### *kup dif* Module

A putative 2549 bp *dif* module containing the *kup* gene (1878 bp) encoding a potassium uptake transporter (Zakharyan and Trchounian, 2001) and an orf of unknown function (210 bp) was first revealed in our previous work (Mindlin et al., 2018). In this work we detected almost identical *kup dif* module in plasmids of three other permafrost strains (Figure 3) and studied its distribution in modern strains. The *kup dif* modules with identity level of 99% were found in plasmids and contigs from *Acinetobacter* strains belonging to different species (Supplementary Table S2). Importantly, seven out of 10 modern plasmids/contigs containing this module were isolated from environmental strains. The origin of the host strains for the remaining three plasmids [CP026424.1; APOG01000001.1; JWHB01000051.1] could not be established. Therefore, this module, which likely controls the uptake of potassium, may increase the fitness of *Acinetobacter* strains living in the environment but not in the clinic.

### *dif* Sites and *dif* Modules in *Acinetobacter* Chromosomes

To reveal whether the presence of multiple *dif* sites is restricted to *Acinetobacter* plasmids, we next analyzed the number and location of *dif* sites in the *Acinetobacter* chromosomes. Usually a single *dif* locus involved in the resolution of chromosomal concatenates is located near the chromosome terminus (Carnoy and Roten, 2009). Surprisingly, we found that the *Acinetobacter* chromosomes are organized in a different way. Besides the main *dif* site (*dif1*), we revealed additional *dif* sites in most completely sequenced circular chromosomes of different species of the *Acinetobacter* genus. In total, we analyzed 20 sequenced chromosomes of 13 *Acinetobacter* species, and only one of them did not contain additional *dif* sites.

As a rule, the number of discovered *dif* sites varied from one to five and they were located far from *dif1* (e.g., *A. lwoffii* WJ10621 [CM001194.1]; *A. lwoffii* ZS207 [NZ_CP019143.1]; *A. johnsonii* XBB1 [CP010350.1]; *A. nosocomialis* SSA3 [CP020588.1]). Interestingly, we found two *Acinetobacter* strains that contained multiple additional *dif* sites in their chromosomes, *Acinetobacter indicus* SGAir0564 with 14 additional chromosomal *dif* sites [CP024620.1] and *Acinetobacter* sp. SWBY1 with 15 additional *dif* sites [CP026616.1].

It should be mentioned that strains containing multiple chromosomal *dif* sites were found occasionally when studying the distribution of different *dif* modules. In particular, the *terC dif* module, almost identical to that from plasmid pALWED1.4 (99% identity), was revealed in the chromosome *of A. indicus* SGAir0564 (CP024620.1). It was flanked by typical p*dif* sites (XerC/D and XerD/C) and was located at 2363397–2364617 bp, far from the main chromosomal *dif1* site at position 1526563. Similarly, we found a variant of the *add dif* module in the chromosome of the *Acinetobacter* sp strain SWBY1 [CP026616.1].

### Comparative Analysis of Plasmid and Chromosomal *dif* Sites

To get insight into the origin and specific structure of the additional chromosomal *dif* sites, we compared their sequences with the main *dif1* sites from *Acinetobacter* chromosomes and with p*dif* sites from *A. baumannii* and *A. lwoffii* (see Materials and Methods). In total 109 p*dif* sites from 23 plasmids of *A. lwoffii*, 115 p*dif* sites from 33 plasmids of *A. baumannii*, 20 main *dif1* and 40 additional chromosomal *Acinetobacter dif* sites were analyzed.

**FIGURE 4:**
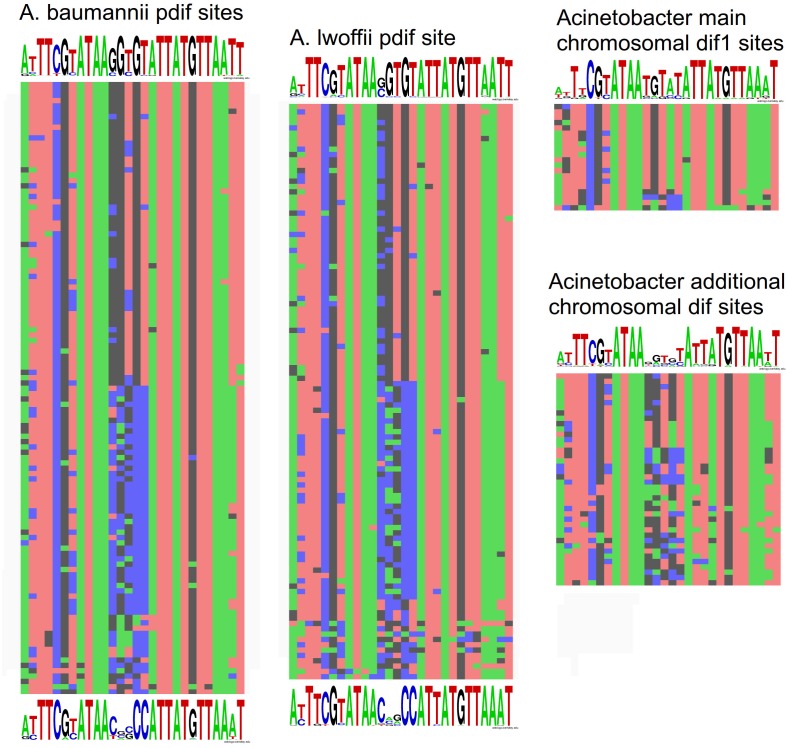
Nucleotide variability within *dif* sites in *Acinetobacter* plasmids and chromosomes. All *dif* sequences analyzed in this study are presented in Supplementary Table S1.

All main features of classical *dif* sites were revealed in all studied groups (Figure 4). All of them have a length of 28 bp and are composed of two 11 bp regions comprising the binding sites for XerD (more conserved) and XerC (less conserved), separated by a more variable 6 bp central region. The inner parts of the XerC and XerD binding motifs contain inverted repeats forming a palindrome and are highly conserved while their outer regions are more variable (Figure 4).

Comparison of *dif* sites from different groups revealed that (Figure 4): (i) p*dif* sites from plasmids of distant species *A. baumannii* and *A. lwoffii* (*A. pseudolwoffii*) are very close to each other in their consensus structure and form two clusters with different consensus sequences of five central nucleotides; (ii) additional chromosomal *dif* sites differ significantly in their structure from the main chromosomal *dif1* site, especially in their central part, while (iii) they are more similar to the plasmid p*dif* sites from both clusters. It should be mentioned that the structure of the main chromosomal *dif1* sites of *Acinetobacter* corresponds well to the consensus of *dif* sites from Proteobacteria chromosomes (Carnoy and Roten, 2009). Thus, it can be hypothesized that the plasmid p*dif* sites and additional chromosome *dif* sites have originated from a common genetic element, whose identity remains to be established.

## Discussion

The results of this and previous studies (D’Andrea et al., 2009; Merino et al., 2010; Grosso et al., 2012; Feng et al., 2016; Blackwell and Hall, 2017; Cameranesi et al., 2018; Hamidian and Hall, 2018) revealed the common presence of several (multiple) copies of *dif* sites and associated *dif* modules in plasmids and chromosomes of various *Acinetobacter* species including pathogens, commensals, and free-living bacteria belonging to different phylogenetic branches of the genus. Neither the additional copies of *dif* sites nor the mobile elements containing them are commonly found in other bacteria, even closely related to *Acinetobacter*, suggesting that their presence is a characteristic feature of the *Acinetobacter* genus. It should be noted that previous studies were mainly limited to the description of *dif* modules with antibiotic resistance genes (D’Andrea et al., 2009; Merino et al., 2010; Grosso et al., 2012; Povilonis et al., 2013; Blackwell and Hall, 2017). It was proposed that their distribution depends on the action of the site-specific recombination system *dif*/Xer. At the same time, the origin of the additional copies of *dif* sites, the mechanisms of their distribution and their role in the bacterial genome have not been studied in detail.

The results obtained in this study suggest that multiple *dif* sites present in *Acinetobacter* plasmids allow the formation of simple mobile elements containing various adaptive genes. In particular, analysis of the plasmid genomes from the ancient *A. lwoffii* (*A. pseudolwoffii*) strains revealed novel *dif* modules encoding different adaptive functions, including the resistance to chromium (*chrAB dif*, Mindlin et al., 2018), tellurium (*terC dif*) and organic peroxides (*ohr1 dif*), potassium (*kup dif*) and sulfate (*sulP dif*) transporters, toxin-antitoxin systems (*add dif*). All the detected genetic elements also have almost identical copies in the plasmids of modern *Acinetobacter* strains belonging to different species. It should be emphasized that the *dif* modules described in the present work were revealed in plasmids from only five studied permafrost strains of *A. lwoffii* group, and many more *dif* modules containing other adaptive genes are likely to be found in the future.

Based on their distribution, three groups of known *dif* modules can be distinguished: (i) mobile elements that encode metabolic and regulatory functions (e.g., resistance to chromium and tellurium, potassium uptake) and ensure survival under natural conditions, and mainly found in plasmids of environmental strains of *Acinetobacter* (described in this study); (ii) mobile elements found in both clinical and natural strains (including *dif* modules encoding sulfate permease, organic hydroperoxide resistance and toxin/antitoxin protein pairs, described in this study); (iii) mobile elements containing genes of antibiotic resistance and found almost without exception in plasmids of clinical strains of *Acinetobacter*, mainly *A. baumanni* (D’Andrea et al., 2009; Merino et al., 2010; Grosso et al., 2012; Blackwell and Hall, 2017). It is also appropriate to place in this group an *Acinetobacter* module encoding a putative virulence factor (Sel1-repeat protein) described by Lean and Yeo (2017). According to our preliminary data, it is flanked by XerC/D and XerD/C sites and is widely distributed in *A. baumannii* plasmids, similarly to classical *dif* modules.

Despite their different distribution, comparative molecular analysis of the *dif* modules described in this work and those containing antibiotic resistance genes described previously (D’Andrea et al., 2009; Merino et al., 2010; Grosso et al., 2012; Povilonis et al., 2013; Blackwell and Hall, 2017) suggests that they belong to the same group of unique genetic elements characteristic to the genus *Acinetobacter.* The wide distribution of such modules among plasmids of various *Acinetobacter s*pecies indicates their ability to transfer horizontally and suggests that they form a special group of mobile genetic elements.

We demonstrated that multiple *dif* sites and *dif* modules can also be found in most *Acinetobacter* chromosomes. The main *dif1* site in the terminus region of the chromosome is required for the resolution of chromosome dimers by the XerC/XerD system and if it is transferred to other regions of the chromosome the process of dimer resolution is impaired, as shown for *Escherichia coli* (Cornet et al., 1996; Pérals et al., 2000). Thus, additional *dif* sites present in the *Acinetobacter* chromosomes are unlikely to function in the dimer resolution. Moreover, analysis of their sequences revealed that they are more closely related to p*dif* sites found in plasmids from various *Acinetobacter* species than to the chromosomal site *dif1*. Several examples of *dif* modules were also found in *Acinetobacter* chromosomes (this work). Thus, it can be proposed that the additional chromosomal *dif* sites may participate in the movement of *dif* modules encoding antibiotic resistance and various adaptive functions from plasmids to the chromosome and backward. Indeed, a *dif* module with the *Acinetobacter*-specific carbapenemase gene OXA-58 was recently found inserted into the XerC/XerD site of a prophage in the *Proteus mirabilis* chromosome (Girlich et al., 2017). As far as we know this is the first description of an XerC/XerD-dependent insertion of *Acinetobacter* antibiotic resistance genes within a bacterial chromosome.

It is natural to assume that the common role of all types of *dif* modules is to increase the fitness of their respective bacterial hosts in their habitats. Our results suggest that the *dif* modules possess the properties of mobile genetic elements capable to translocate between plasmids and chromosomes in *Acinetobacter* strains belonging to different species. Additional research is highly needed to assess the vast diversity of *dif* modules in this clinically important genus and reveal their contribution to horizontal gene transfer and plasmid variability.

## Author Contributions

AM, AB, and MP conducted the sequencing and assembly of plasmids. All authors performed the bioinformatic analysis. MP performed the experiments. SM, MP, and AM wrote the manuscript. All authors contributed to the discussion and provided comments on the manuscript.

## Conflict of Interest Statement

The authors declare that the research was conducted in the absence of any commercial or financial relationships that could be construed as a potential conflict of interest.

## Footnotes

1 https://apps.szu.cz/anemec/Genomes.pdf

2 http://unipro.ru

3 https://weblogo.berkeley.edu/logo.cgi

## Supplementary Material

TABLE S1 Distribution of *dif* sites in *Acinetobacter* plasmids and chromosomes.

TABLE S2 Distribution of *dif* modules in ancient (permafrost) and modern *Acinetobacter* strains.
