# Supplementary material for: Adaptive dif Modules in Permafrost Strains of Acinetobacter lwoffii and Their Distribution and Abundance Among Present Day Acinetobacter Strains
Source: Front Microbiol. 2019 Mar 29;10:632. doi: 10.3389/fmicb.2019.00632 (PMC6449649; doi:10.3389/fmicb.2019.00632)
Supplement: TABLE S2 — Distribution of dif modules in ancient (permafrost) and modern Acinetobacter strains. [file Table_2.docx]

**Supplement**

**Table S2. Distribution of *dif* modules in ancient (permafrost) and modern**

***Acinetobacter* strains**

| **Dif module** | **Ancient plasmid /position** | **Modern plasmid/AC** | **Contigs from modern strains/AC** |
| --- | --- | --- | --- |
| ***chrAB**** | pALWED1.3/ 3-3013  pALWED3.5/9684-12694  pALWEK1.5/ 1-605+5819-8227 | *A. schindleri* ACE p5AsACE [CP015620.1] *A*. sp. M131 pM131-6 [JX101643.1] | *A .lwoffii* NCTC 5866 [[AIEL01000040.1](https://www.ncbi.nlm.nih.gov/nuccore/APQS01000013)]  *A. lwoffii* NIPH 478 [[APQU01000026.1](https://www.ncbi.nlm.nih.gov/nuccore/APQS01000013)]  *A. lwoffii* ATCC 9957 [APQT01000010.1]  *A. nosocomialis* UBA2046 [DDAY01000090.1]  *A. lwoffii* MII [JQCU01000331.1]  *A. indicus* KM7 [JZRF01000028.1]  *A. baumannii* TG02011 [ASES01000041.1]  *A. pittii* 573719 ab573719 [JFYA01000016.1]; *A*. sp. UBA3450 [DFRZ01000163.1]; *A*. sp. UBA3461 [DFRO01000013.1] |
| ***terC*** | pALWED1.4/ 10831-12052  pALWED2.1/ 23177-24346+52*  pALWED3.6/74538-75760  pALWEK1.1/ 172447-173668 | *A. lwoffii* ZS207 pmZS [CP019144.1]  *A*. *variabilis* ACNIH1, pAC1-148e [CP026424.1] | *A. baumannii* ABUH794 [[LZWX01000112.1](https://www.ncbi.nlm.nih.gov/nuccore/LZWX01000112)],  *A. nosocomialis* UBA2046 [DDAY01000087]  *A. lwoffii* MII [JQCU01000202.1].  *A. schindleri* ACNIH3 NODE_66  [[PQLP01000066.1](https://www.ncbi.nlm.nih.gov/nuccore/PQLP01000066)] |
| ***add*** | pALWED2.1/ 103484-104251  pALWED2.3/ 20268-21035  pALWED3.6/167930-167217  pALWEK1.1/ 164115-164882 | *A. lwoffii* ZS207 pmZS [CP019144.1];  *A .baumannii* MAD, plasmid unnamed; partial sequence [[AY665723.1](https://www.ncbi.nlm.nih.gov/nucleotide/AY665723?report=genbank&log$=nuclalign&blast_rank=3&RID=FK2WTUDT015)]  *A.baumannii* WCHAB005133 plasmid pOXA58_005133 [[CP026749.1](https://www.ncbi.nlm.nih.gov/nucleotide/CP026749?report=genbank&log$=nuclalign&blast_rank=6&RID=FK2WTUDT015)]; *A. johnsonii* XBB1 plasmid pXBB1-9 [CP010351.1] | *A .lwoffii* NCTC 5866 [[APQS01000013.1](https://www.ncbi.nlm.nih.gov/nuccore/APQS01000013)]  *A. lwoffii* NIPH 478 [[APQU01000004.1](https://www.ncbi.nlm.nih.gov/nuccore/APQS01000013)]  *A. lwoffii* MII [JQCU01000243.1]  *A. towneri* KCTC 12419 [BBNL01000051.1] |
| ***ohr1***  ***ohr2*** | pALWED1.2/ 35721-36783  pALWVS1.1/132248-133310  pALWEK1.1/204826-205888  not found | not found  *A. baumannii* 1906, pMAC/ 5084-6151 [AY541809.1];  *A. baumannii* AR_0070, plasmid unnamed 2 [CP027180]; *A. baumannii* AR_0052, plasmid unnamed 4 [CP027186]; *A*. *variabilis* ACNIH1, pAC1-148e [CP026424.1] | *A. radioresistens* UBA4041 [DGAB01000004.1]; *A. gerneri* KCTC12415 [BBLI01000074.1]; *A. schindleri* NIPH900 [APPI01000008.1]; *A. haemolyticus* ATTC19194 [ADMT01000026.1]*; A. baumannii* JX101 [QCXU01000042.1]; *A. pittii* PR348 [NGEJ01000015.1] and 13 additional  *Acinetobacter* *pseudolwoffii* ANC 5347 [[PGOZ01000028.1](https://www.ncbi.nlm.nih.gov/nuccore/PGOZ01000028)];  *Acinetobacter* *radioresistens* 869535 [[JEXH01000071.1](https://www.ncbi.nlm.nih.gov/nuccore/JEXH01000071)];  *A. baumannii* ANC 4097 [[APRF01000020.1](https://www.ncbi.nlm.nih.gov/nuccore/APRF01000020)];  *A. baumannii* PR384 [[NGER01000067.1](https://www.ncbi.nlm.nih.gov/nuccore/NGER01000067)];  *Acinetobacter* *indicus* CIP 53.82 [[APRK01000007.1](https://www.ncbi.nlm.nih.gov/nuccore/APRK01000007)]; *A.bereziniae* 507_ABAU [[JVEK01000235.1](https://www.ncbi.nlm.nih.gov/nuccore/JVEK01000235)] |
| ***sulP*** | pALWED1.3/5460-7618 | *A.lwoffii* ZS207 pZS-3 [CP019145.1]  *A.lwoffii* ZS207 pmZS [CP019144.1] | *A. lwoffii* NIPH 715 [[APOT01000008.1](https://www.ncbi.nlm.nih.gov/nuccore/APOT01000008)]  *A. johnsonii* Aj2199 [LVIB01000061.1]  *A. lwoffii* CIP64.7 [[APRY01000056.1](https://www.ncbi.nlm.nih.gov/nuccore/AMZS01000024)]  *A.* *lwoffii* CIP51.11 [[APRU01000010.1](https://www.ncbi.nlm.nih.gov/nuccore/FZRG01000055)]  *A*. *lwoffii* AR2-3 [[MJIQ01000063.1](https://www.ncbi.nlm.nih.gov/nuccore/APPQ01000017)] |
| ***sulP-upsA*** | pALWEK1.10/3879-6617 | *A.baumannii* WCHAB005133, pOXA58_005133 [CP026749.1];  *A. johnsonii* XBB1, pXBB1-6 [CP010357.1];  *A.pittii* WCHAP005069, p2_005069 [CP026088.1] | *A. johnsonii* AJ01M [FZRG01000055.1]  *A. schindleri* CIP107287 [APPQ01000017.1]  *A.pittii* PR339 [NGEB01000088.1]  *A.baumannii* PR352 [NGCF01000042.1],  *A*. *courvalinii* WC-323 [AMZS01000024.1];  *A. lwoffii* CIP 101966 [APRX01000008.1];  *A*. sp. (Taxon 21) ANC 3929 [APRH01000002.1] |
| ***kup*** | pALWED1.4/ 7097/9645 pALWED2.1/ 117351-119899 pALWED3.5/14047-16568+27*;  pALWEK1.4/365-2913 | *A.lwoffii* ZS207 pmZS [CP019144.1]  *A*. *variabilis* ACNIHI, pAC1-148e [CP026424.1] | *A.lwoffii* NBRC 109760 [BBSQ01000035.1];  *A. indicus* KCTC 42012 [NEXW01000023.1]  *A. schindleri* DRN [QCWW01000055.1]  *A. lwoffii* NCTC 5866 [[AIEL01000035.1](https://www.ncbi.nlm.nih.gov/nuccore/APQS01000013)]  *A*. *lwoffii* CIP162 [APOG01000001.1];  *A*. *towneri* YT-02 [NOZT01000067.1];  *A*. *variabilis* YZS-X1-1 [JWHB01000051.1];  *A*. sp. ACNIH4 [PQLO01000073.1] |

*previously was described in Mindlin et al., (2018)
